# Aluminum

**Published:** 1869-11

**Authors:** 


ALUMINUM.—The scientific editor of the *Independent* says: In his *Journal of Applied Chemistry*, Professor Joy says that a few years ago a pound of aluminum could not have been purchased for $200, and even at that price there were few manufacturers hardy enough to take the order. At the present time it can be readily manufactured for seventy-five cents, if not for fifty cents a pound; and the probabilities are that we shall soon be able to obtain it for a quarter of a dollar. Its principal use thus far is in the manufacture of aluminum bronze, or alloy of copper with ten per cent. of aluminum, which possesses remarkable strength, tenacity and elasticity.

Aluminum will prove a very important and useful metal; useful in the manufacture of surgical instruments and appliances, etc., and we are glad that it has at last reached the inevitable point of a material reduction in price.

